# Comorbidity and cost burden among ischemic stroke inpatients aged 60 years and older in middle-high-income region in China: a multicenter cross-sectional study

**DOI:** 10.3389/fpubh.2025.1692057

**Published:** 2025-12-18

**Authors:** Runda Jiao, Hongyu Ma, Shan Gao, Yue Yang, Tianyi Zhang, Lihua Liu

**Affiliations:** 1 PLA Medical School, PLA General Hospital, Beijing, China; 2 Department of Medical Innovation and Research, PLA General Hospital, Beijing, China; 3 Neurovascular Center, Changhai Hospital, Naval Medical University, Shanghai, China; 4 Department of Endocrinology, First Medical Center of PLA General Hospital, Beijing, China; 5 Department of Nephrology, First Medical Center of PLA General Hospital, Beijing, China; 6 State Key Laboratory of Kidney Diseases, Beijing, China

**Keywords:** ischemic stroke, comorbidity, healthcare cost, older population, disease cluster

## Abstract

**Background:**

Older adults with ischemic stroke (IS) are prone to develop comorbidities, thus worsening clinical outcome and intensifying cost burden. Limited studies have revealed evidence linking types of combined diseases with economic burden in IS patients. In this study, prevalent combined diseases and clusters of comorbidity among IS patients aged≥ 60 years were identified. Meanwhile, we explored the combined diseases significantly correlated with incremental hospital costs, aiming to promote the individualized and comprehensive management of IS patients.

**Methods:**

The study was a multicenter, cross-sectional study based on clinical data of IS patients (aged ≥60 years) obtained from three tertiary centers of PLA General Hospital between 2018 and 2023. Patients were stratified into three age groups: 60–69 years, 70–79 years, and ≥80 years. Descriptive analyses were performed to show patient number, the composition of combined diseases, and medical costs. Apriori association rules mapped the clusters of comorbidity. Spearman correlation analysis combined with age-stratified quantile regression identified cost-intensive health conditions.

**Results:**

Apriori correlation analysis revealed a dominant cardio-metabolic-cluster and the intermediary role of diabetes. Hypoproteinemia aggregated with pulmonary infection and anemia, forming a clinically significant malnutrition-infection-anemia triad. The healthcare costs were highest in advanced older adult group despite an overall expenditure declining from 2018 to 2023. Spearman correlation and quantile regression analyses showed correlation between incremental costs and malnutrition-infection-anemia triad, especially at higher cost quantiles. Pulmonary infection was associated with relatively higher cost burdens in patients aged ≥80 years, with significant estimated increases of about ¥5,953, ¥8,538, ¥13,810, and ¥18,945 at the 10th, 25th, 50th, and 75th percentiles. Hypoproteinemia tended to correspond to the significant rise in costs at the 50th percentile for patients aged 60–69 years (*β* = ¥20,957) and for those aged ≥80 years (*β* = ¥12,962).

**Conclusion:**

This study identified three prevalent comorbidity clusters in the study population: cardio-metabolic, diabetes-mediated multi-organ injury, and malnutrition-infection-anemia clusters. From the perspective of healthcare cost, pulmonary infection, hypoproteinemia, and anemia might represent the principal cost-intensive clusters of comorbidity, especially in those aged ≥80 years. The persistent cost-age gradient necessitates risk-stratified resource allocation. Implementation of geriatric-specific comorbidity control protocols, particularly targeting the identified clusters with high costs, may optimize both clinical outcomes and healthcare economics in aging populations.

## Introduction

Nowadays, stroke persists as a preeminent global health challenge, imposing a substantial burden on healthcare system worldwide. Previous studies on global burden of diseases revealed that stroke is the second-leading cause of death globally (approximately 6.55 million deaths annually) and a primary contributor to disability-adjusted life years (DALYs) (1–3). China has been faced with advanced population aging burden, exacerbating the incidence of stroke. There are approximately 3.4 million new cases annually, and stroke accounts for 19.9% of the total mortality (4). Furthermore, over half of stroke survivors experience varying degrees of functional or cognitive impairment, imposing a substantial health and economic burden on families and society (5, 6).

Ischemic stroke (IS) is the most common subtype of stroke, accounting for over 80% of all stroke cases (7). Due to age-related decline in physiological reverse and organ function, older adults with IS are prone to develop comorbidities, thus may further worsening the clinical outcome and intensifying the economic burden of IS patients (8, 9). It is well-established that multimorbidity, defined as the co-existence of 2 or more chronic health conditions in an individual, is highly prevalent in geriatrics. Core diseases and their comorbidities encompass interactions between physical, mental, and functional disorders, leading to synergistic health deterioration (10). Previous studies have showed that comorbidities in geriatric IS patients follow distinct, non-random clustering patterns, driven by shared vascular pathobiology and aging-related physiological changes, mainly including cardio-cerebro-metabolic cluster (hypertension, diabetes, ischemic heart disease), neuro-psychiatric cluster (vascular cognitive impairment; depression, anxiety) (11, 12).

Hospitalization costs for geriatric patients with ischemic stroke exhibit significant heterogeneity, largely attributable to multiple factors, including profile of IS, combined diseases, acute interventions (thrombolysis or thrombectomy), intensive care utilization, and extended rehabilitation (13, 14). Previous studies on correlation between medical costs and combined diseases revealed that diabetes mellitus, ischemic heart disease, chronic kidney disease and chronic respiratory diseases showed positive correlation with increased medical costs among the geriatrics (15, 16). However, rare studies have clarified which types of combined diseases impose a substantial economic burden on geriatric patients with ischemic stroke, especially focusing on the difference between various age groups.

In this study, we enrolled multicenter geriatric patients with ischemic stroke from 2018 to 2023, focusing on the compositions and clusters of comorbidity in the study population, aiming to identify common combined diseases and construct comorbidity networks of geriatric IS patients. Meanwhile, we have performed correlation and quantile regression analyses so as to clarify the association between healthcare costs and multiple types of combined diseases in various age groups, providing guidance for the individualized and comprehensive management of geriatric IS patients, ultimately aiming to formulate cost-effective strategies for stroke care.

## Materials and methods

### Study design and participants

The study is a multicenter, cross-sectional study based on clinical data obtained from three affiliated tertiary centers of PLA General Hospital from January 1st, 2018 to December 31st, 2023. Inclusion criteria encompassed patients (aged ≥60 years) with a principal diagnosis of ischemic stroke, and identifiable ischemic lesions confirmed radiologically on brain CT or MRI. Patients were excluded if they were diagnosed as hemorrhagic stroke, pre-existing neurological deficits (mRS >2), transient ischemic attack, severe combined diseases (such as metastatic malignancies, multiple organ failure, or terminal illnesses), or treatment discontinuation due to inter-hospital transfer. This study received exemption from approval by the Institutional Review Board of Chinese PLA General Hospital due to its retrospective analysis of fully de-identified clinical data. Informed consent for clinical data collection and secondary use was obtained from all patients upon admission; therefore, additional informed consent specific to this study was waived. The study was conducted in accordance with the ethical standards of the responsible institutional and regional committees for human research and adhered to the principles outlined in the Declaration of Helsinki.

### Data collection and definition

Clinical data were collected through hospital information system (HIS) of three medical center. The variables collected and analyzed in this study included demographic data (age, gender, body height, body weight, insurance state and region), the profile of IS [major diagnosis at discharge, other diagnoses at discharge, stage of IS and Trial of Org 10172 in Acute Stroke Treatment (TOAST)], medical costs, length of hospital stay and in-hospital outcome (in-hospital death).

The formula for body mass index (BMI) is: BMI = body weight (kg)/body height^2^ (m^2^). Insurance states are classified into full insurance, which covers more than 80% of medical costs, partial insurance (covering less than 80%), and self-paying. Urban and rural regions are defined based on the classification codes of the address issued by the Chinese government. The diagnosis and classification of ischemic stroke were described according to TOAST (17). The combined diseases included in the analyses were collected from all items of other diagnoses at discharge. We have extracted and pooled combined diseases of the ischemic stroke patients and selected top 20 types of the most common combined diseases (the disease should have more than 500 sample size) for further analysis. In this study, the diagnostic criteria for hypoalbuminemia and anemia were as follows: (1) Hypoalbuminemia: a serum albumin level <35 g/L. (2) Anemia: a hemoglobin level <120 g/L for males and <110 g/L for females (18, 19). Moreover, we focused on the medical costs during hospitalization, which were categorized into four categories: drug costs (including all medications administered during the hospitalization), inspection and testing costs (covering diagnostic expenses, imaging, and laboratory tests), supporting treatment costs (such as nursing services, bed fees, blood transfusions, and oxygen therapy), and surgical and other treatment costs (encompassing all invasive procedures performed for therapeutic purposes). We assessed the medical costs from the perspective of the Chinese healthcare system and exhibited the cost using China Yuan (¥). Healthcare costs were not adjusted for inflation as medical service and drug prices remained stable and were recorded in local currency within a fixed set of tertiary hospitals. Length of hospital stay was calculated according to admission date and discharge date. Besides, the in-hospital outcome we concentrated on was in-hospital mortality, defined as death occurring after hospital admission and before discharge. Patients who were transferred to other hospitals with unclear outcomes were excluded.

### Grouping and statistical analysis

To clarify age-specific determinants of combined diseases and healthcare expenditures, the cohort was stratified into three age groups in our study: 60–69 years, 70–79 years, and ≥80 years. Categorical variables were expressed as frequency (percentage) and were analyzed with *χ*^2^ tests. Continuous variables with non-normal distribution were expressed as median and interquartile range (IQR), and were analyzed with Kruskal–Wallis tests. The proportions of missing data in variables were less than 20%. The patterns of missingness were examined, and no systematic bias was found in the missing data, supporting the validity of the imputation approach. Missing data were handled using the Multiple Imputation by Chained Equations (MICE) method in R studio (Version 4.4.2). Five imputed datasets were generated using predictive mean matching (PMM), in which missing values are replaced with observed values from the closest matching cases based on a regression model. A fixed random seed of 500 was used to ensure reproducibility of the imputation process. Composition and proportion of combined diseases from 2018 to 2023 were shown in the stack bar plot. Changes in total medical costs and in four subtypes of medical costs from 2018 to 2023 were shown in grouped line plots.

Apriori algorithm is a classic technique in association rule mining, widely used to identify significant comorbidity patterns. Three core metrics, support (the prevalence of a disease set within a population), confidence (the conditional probability of co-occurrence) and lift (the strength of a rule by comparing the actual co-occurrence frequency to the expected frequency if the items were independent), were applied to screen and evaluate co-occurrence strength and association of disease pairs (20). The comorbidity network was constructed using Apriori algorithm based on “arules” package in R studio (Version 4.4.2), with a minimum support of 0.02, minimum confidence of 0.35, minimum length of itemsets of 2. The proportions of missing data in variables were less than 20%. The patterns of missingness were examined, and no systematic bias was found in the missing data, supporting the validity of the imputation approach. Missing data were handled using the Multiple Imputation by Chained Equations (MICE) method in R studio (Version 4.4.2). Five imputed datasets were generated using predictive mean matching (PMM), in which missing values are replaced with observed values from the closest matching cases based on a regression model. A fixed random seed of 500 was used to ensure reproducibility of the imputation process. The strength of the association between itemsets was measured by lift values, which are represented by the color gradient in the network plot, with higher lift values shown in darker shades. No threshold was set for the lift values. In addition, Spearman correlation analysis and quantile regressions were carried out to identify the combined diseases most strongly associated with incremental hospitalization costs across the three age groups. A multivariable quantile regression model, incorporating all comorbidities, was constructed to assess incremental costs at the 10th, 25th, 50th, 75th, and 90th cost percentiles (*τ* = 0.1, 0.25, 0.50, 0.75, 0.9), while adjusting for demographic variables including gender, BMI, region, insurance, admission stage, TOAST, and length of hospital stay. Coefficient trajectories were derived from age-stratified models in which each comorbidity was analyzed separately at each 10 percentiles, with the same adjustment set. Ninety-five percent confidence intervals were obtained from 200 bootstrap replications. Multiple testing corrections was performed using the False Discovery Rate (FDR) adjustment with Benjamini–Hochberg method to prevent Type I errors in the comparisons across 20 comorbidities and three age groups. Moreover, sensitivity analysis based on population excluding those hospitalized in 2020–2021 has been performed to justify the potential confounding effects by COVID-19 pandemic on the healthcare system.

## Results

### Study population and baseline characteristics

**Figure 1:**
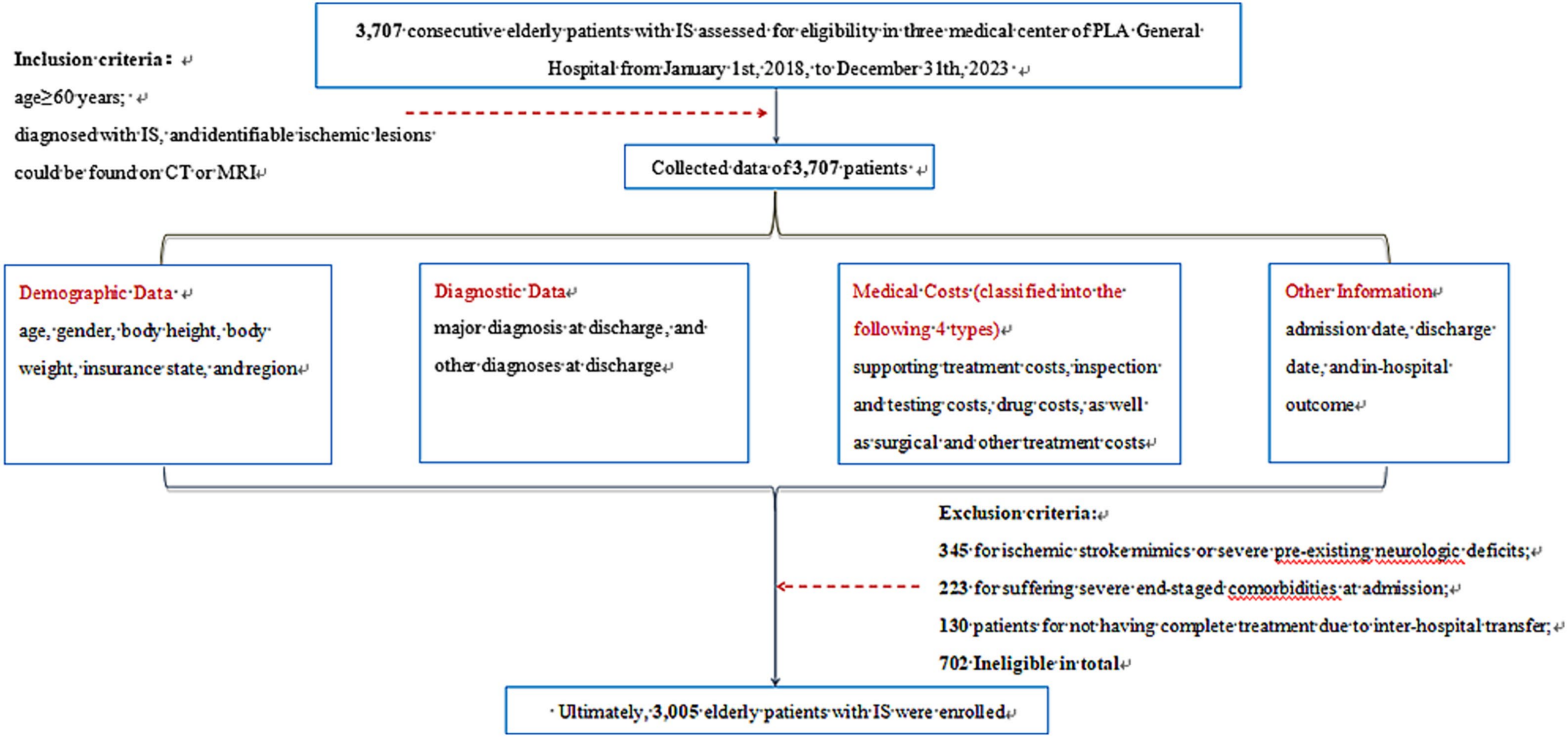
Eligibility of the study population. The flowchart of the eligibility of 3,005 patients. IS, ischemic stroke; CT, computerized tomography, computed tomography; MRI, magnetic resonance imaging.

From January 1st, 2018, to December 31st, 2023, a total of 3,703 patients aged ≥60 years hospitalized in three tertiary hospitals were assessed for eligibility. Ultimately, 3,005 patients were enrolled according to inclusion and exclusion process (Figure 1). The number of patients, shown in Table 1, fluctuated over the years from 2018 to 2023, reaching its nadir in 2020 and peaking in 2022. Patients aged ≥80 years constituted the largest proportion from 2018 to 2020, with a decline in 2021 potentially associated with COVID-19 pandemic.

**Table 1:** The numbers of enrolled IS patients in various age groups from 2018 to 2023.

Year	60–69 years (*n* = 936)	70–79 years (*n* = 934)	≥80 years (*n* = 1,135)	Counts in each year
2018	131	123	194	448
2019	106	107	156	369
2020	96	110	139	345
2021	185	185	218	588
2022	225	215	245	685
2023	193	194	183	570

IS, ischemic stroke.

Demographic data were shown in Table 2. The number of male patients predominated in all age groups, but the proportion of males decreased with age: 67.3, 60.0, and 53.0% in 60–69, 70–79, and ≥80 age groups, respectively (*p* < 0.001). BMI was slightly lower in ≥80 years group compared to the other groups [23.88 (21.69–25.93) vs. 24.49 (22.21–26.60) vs. 24.42 (22.43–26.42), *p* < 0.001]. Most patients were urban residents, with a higher proportion in the older age groups (65.3% vs. 72.8% vs. 91.6%, *p* < 0.001). In terms of insurance coverage, 38.1% of patients ≥80 years had insurance covering over 80% of medical costs, compared to lower proportions in younger age groups. Although the majority of patients were admitted at acute stage, approximately one-third of the patients were admitted at post-acute stage among all age groups. Regarding stroke subtypes based on TOAST, the proportion of large-artery atherosclerosis (LAA) decreased with age (26.3, 20.6, and 14.5%, *p* < 0.001), while cardioembolism (CE) increased (18.3, 27.3, and 41.1%, *p* < 0.001). Patients in ≥80 years group are prone to have longer hospital stays [14 (11–20) days vs. 15 (11–23) days vs. 17 (12–30) days, *p* < 0.001]. Meanwhile, the in-hospital mortality was significantly higher in ≥80 years group compared to younger groups (1.4% vs. 3.0% vs. 10.2%, *p* < 0.001).

**Table 2:** Baseline characteristics of geriatric ischemic stroke patients in three age groups.

Characteristics	60–69 years (*n* = 936)	70–79 years (*n* = 934)	≥80 years (*n* = 1,135)	*p*-value
Demographics				
Age, years	65 (63, 68)	74 (72, 77)^*^	85 (82, 89)^*#^	*p* < 0.001
Male, *n* (%)	630 (67.3)	560 (60.0)^*^	602 (53.0)^*#^	*p* < 0.001
BMI, kg/m^2^	24.42 (22.43, 26.42)	24.49 (22.21, 26.60)	23.88 (21.69, 25.93)^*#^	*p* < 0.001
Insurance, *n* (%)				*p* < 0.001
Insurance covering >80%	54 (5.8)	129 (13.8)^*^	433 (38.1)^*#^	
Partial insurance	750 (80.1)	709 (75.9)^*^	650 (57.3)^*#^	
No insurance	132 (14.1)	96 (10.3)^*^	52 (4.6)^*#^	
Region, *n* (%)				*p* < 0.001
Urban	612 (65.3)	680 (72.8)^*^	1,040 (91.6)^*#^	
Rural	324 (34.6)	254 (27.1)^*^	95 (8.3)^*#^	
Profile of ischemic stroke				
Stage, *n* (%)				*p* = 0.075
Acute	634 (67.7)	635 (67.9)	759 (66.9)	
Post-acute	302 (32.3)	299 (32.1)	376 (33.1)	
TOAST, *n* (%)				*p* < 0.001
SUE	411 (43.9)	390 (41.8)	361 (31.8)^*#^	
SOE	39 (4.2)	21 (2.2)^*^	33 (2.9)	
LAA	246 (26.3)	192 (20.6)^*^	165 (14.5)^*#^	
SVA	69 (7.4)	76 (8.1)	110 (9.7)	
CE	171 (18.3)	255 (27.3)^*^	466 (41.1)^*#^	
Medical cost, ¥				
Total cost, ¥	25,256 (16,852, 44,723)	25,911 (17,025, 50,893)	35,684 (19,335, 77,994)^*#^	*p* < 0.001
Drug cost, ¥	9,081 (5,047, 16,460)	9,279 (5,296, 9,222)	12,924 (6,381, 32,165)^*#^	*p* < 0.001
Inspection and testing cost, ¥	8,149 (5,978, 12,157)	8,358 (5,817, 13,340)	9,677 (6,115, 17,256)^*#^	*p* < 0.001
Supporting treatment cost, ¥	1,762 (1,203, 2,740)	1,926 (1,118, 3,192)	2,221 (1,319, 4,457)^*#^	*p* < 0.001
Surgical and other treatment cost, ¥	3,733 (2,193, 8,677)	4,352 (2,349, 10,496)	6,495 (2,890, 18,197)^*#^	*p* < 0.001
Length of hospital stay, days	14 (11, 20)	15 (11, 23)^*^	17 (12, 30)^*#^	*p* < 0.001
In-hospital death, *n* (%)	13 (1.4)	28 (3.0)^*^	116 (10.2)^*#^	*p* < 0.001

BMI, body mass index; TOAST, Trial of Org 10172 in Acute Stroke Treatment; SUE, stroke of undetermined etiology; SOE, stroke of other determined etiology; LAA, large-artery atherosclerosis; SVA, small-vessel occulsion; CE, cardioembolism. Continuous variables with non-normal distribution were presented as median and interquartile range (IQR), while categorical variables were presented as frequency (percentage). Non-parametric tests were used for inter-group comparisons of continuous variables, while *χ*^2^ tests were used for component comparisons of categorical variables. *p* < 0.05 were considered statistically significant. ^*^Represents the difference between 70–79 years group and 60–69 years group is significant or the difference between ≥80 years group and 70–79 years group is significant. ^#^Represents the difference between ≥80 years group and 60–69 years group is significant.

### Composition of comorbidities in geriatric IS patients

**Figure 2:**
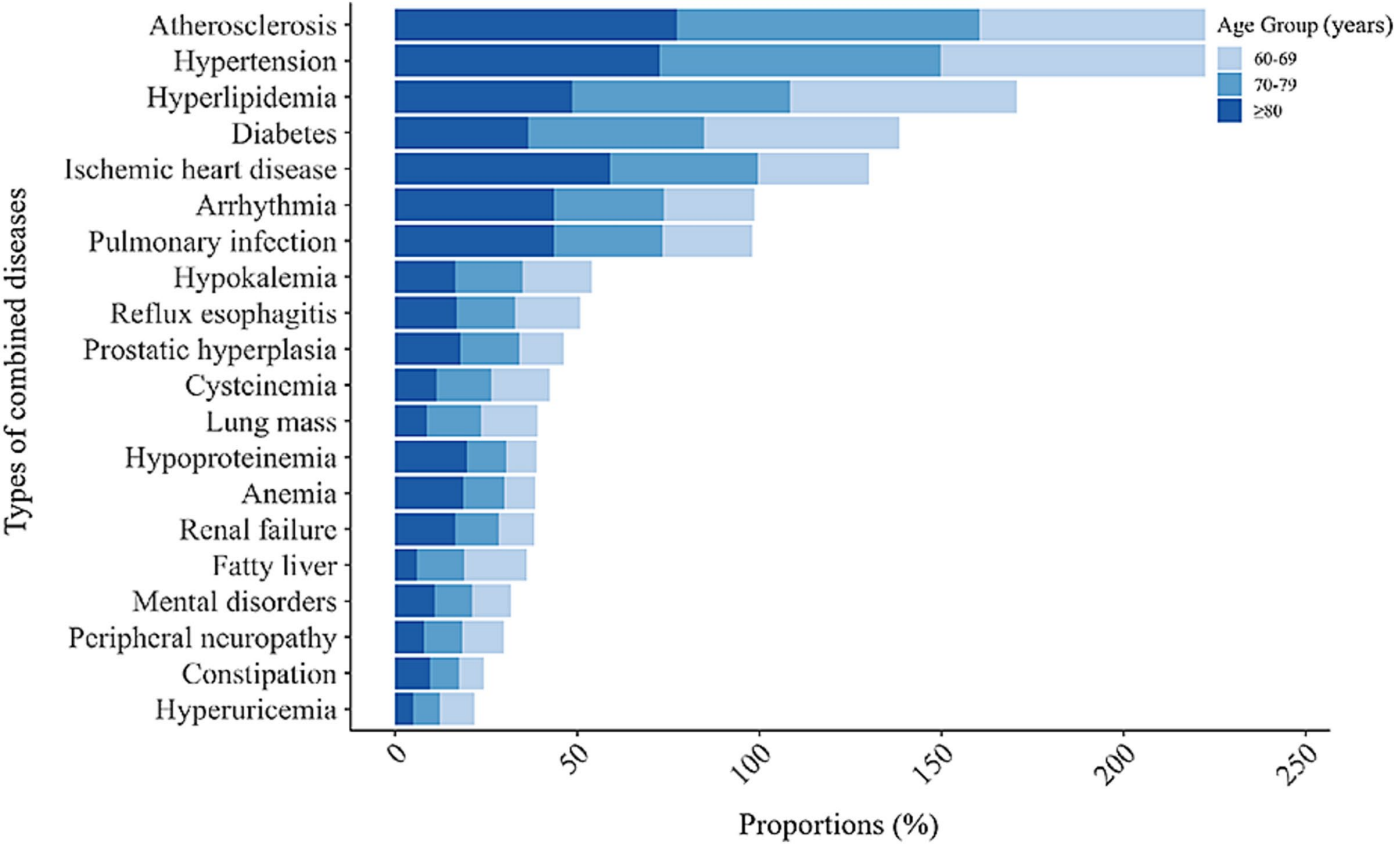
Composition and proportion of combined diseases in the geriatric patients with IS. This figure showed the composition and proportion of the top 20 combined diseases in the study population, displaying in age groups. IS, ischemic stroke.

By analyzing all items of diagnosis in geriatric patients with IS, we have found the most common combined diseases ranking top 20, among which, atherosclerosis, hypertension, hyperlipidemia, diabetes, ischemic heart disease, arrhythmia, pulmonary infection, hypokalemia, reflux esophagitis and prostatic hyperplasia are the top 10 diseases. The proportions of common combined diseases indicated that atherosclerosis, hypertension, and hyperlipidemia affected over half of the study population. Younger patients exhibited higher rates of hyperlipidemia and diabetes, while the patients aged ≥80 years had higher rates of ischemic heart disease, arrhythmias, and pulmonary infection (Figure 2).

**Figure 3:**
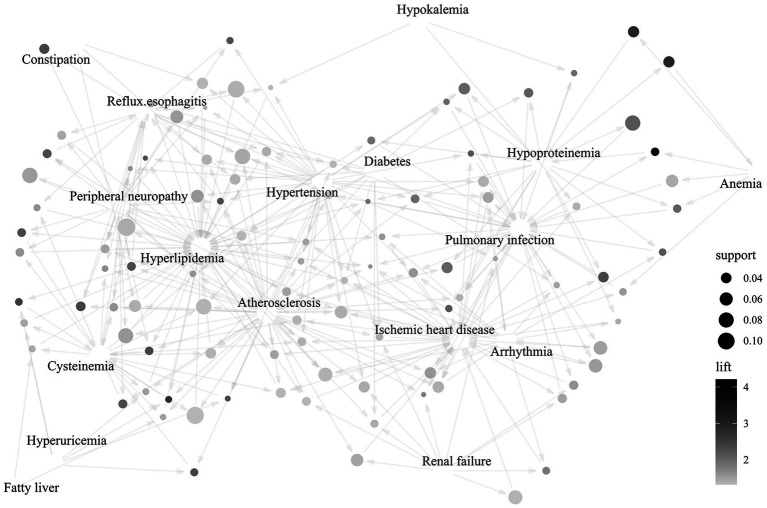
The clusters of comorbidity in the geriatric patients with IS. The clusters illustrated the interrelationships among combined diseases in the study population. Each node represents a connection between distinct combined disease, with node size corresponding to supporting strength, namely, the frequency of a specific pattern in the dataset. The directed edges indicate association rules identified by Apriori, and the arrowheads suggest the direction of these relationships (from antecedent to consequent). Colors range from light gray to black signify increasing lift values, whereby a higher lift denotes a stronger, potentially more clinically significant association between combined diseases.

Apriori correlation analysis revealed a dense network of combined diseases in the study population (Figure 3). A dominant cardio-metabolic cluster composed of atherosclerosis, hyperlipemia, hypertension, and ischemic heart disease exhibited high joint support and lift. Diabetes functioned as a pivotal bridging node that may amplify the co-occurrence of renal failure, peripheral neuropathy, and hypoproteinemia, implicating diabetes as an intermediary factor for multi-organ injury. Hypoproteinemia aggregated with pulmonary infection and anemia, forming a malnutrition-infection-anemia triad of clinical relevance. Although the prevalence of hypokalemia is relatively low in the study population, its strong linkage with arrhythmia and renal failure makes it a possible early marker of future cardio-renal instability. Gastrointestinal issues such as constipation and reflux esophagitis were frequent yet peripheral in the study population, exerting little influence on other combined diseases. These findings support integrated monitoring of cardiovascular, metabolic, and nutritional factors in therapeutic strategy and secondary prevention among geriatric patients with stroke. In addition, we explored the variation of comorbidity clusters between 2018 and 2023.

### Cost burden and related comorbidities

**Figure 4:**
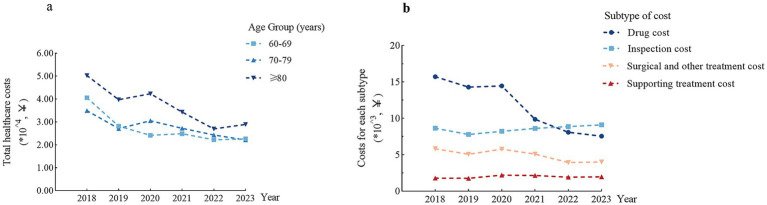
Variation of healthcare costs from 2018 to 2023. **(a)** Variation of total healthcare costs in three age groups from 2018 to 2023. **(b)** Variation of each subtype of healthcare costs from 2018 to 2023.

Hospitalization costs demonstrated significant age-dependent escalation, the medians of healthcare costs were shown in Table 2. ¥25,256 (¥16,852, ¥44,723) for patients aged 60–69 years, ¥25,911 (¥17,025, ¥50,893) for those aged 70–79 years, and ¥35,684 (¥19,335, ¥77,994) for those aged ≥80 years. Annual healthcare costs for all patients decreased from 2018 to 2023, with the greatest reduction observed in patients aged ≥80 years, showing a more than 50% decrease in healthcare costs over 5 years (Figure 4a). Subcomponent analyses revealed substantial progressive declines in drug costs while diagnostic fees, procedural costs, and supportive care fees remained temporally stable (Figure 4b).

**Figure 5:**
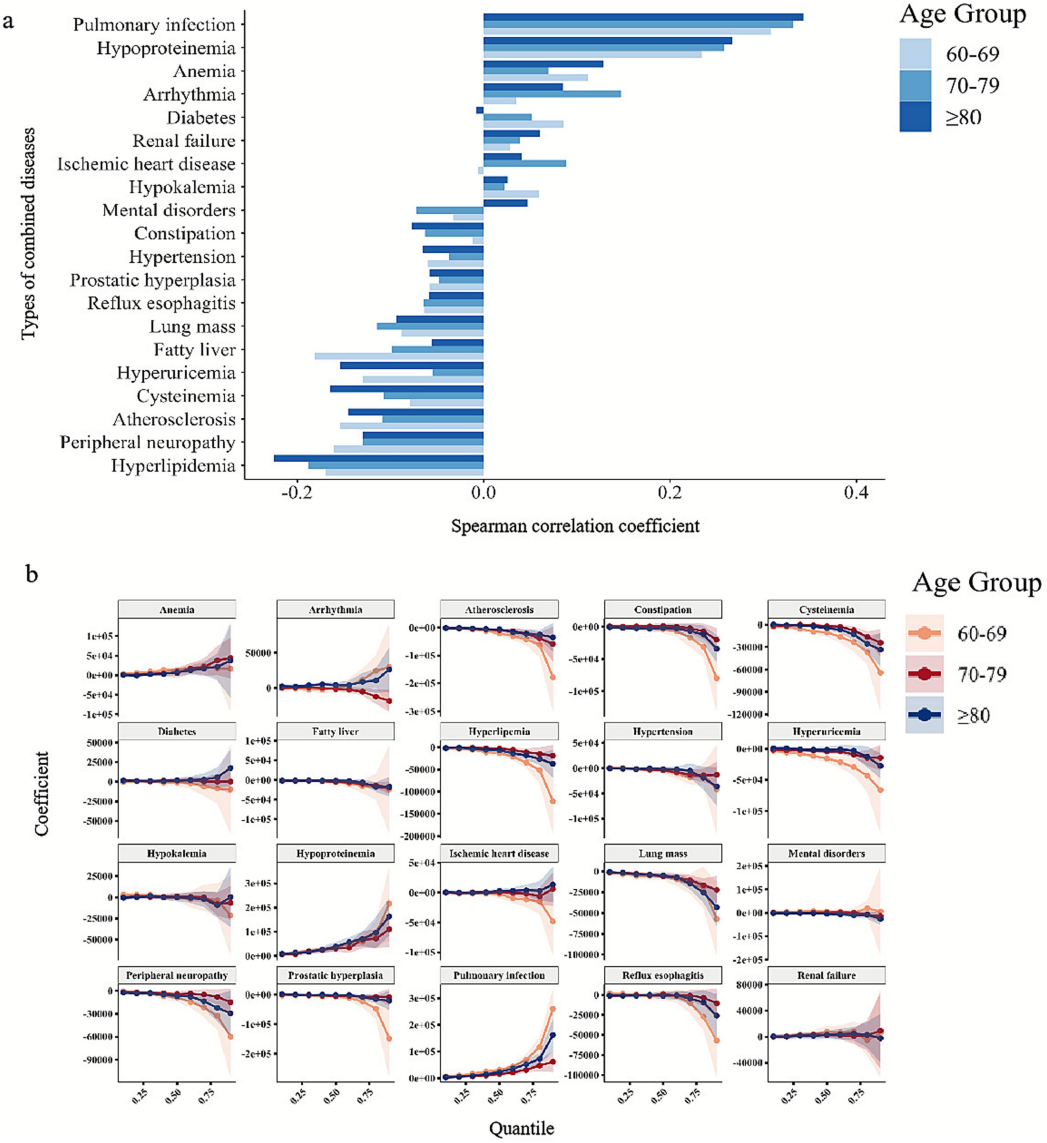
The relationship between combined diseases and healthcare costs. **(a)** Spearman correlation analysis between combined diseases and healthcare costs by age group. Spearman correlation analysis revealed the association between the presentation of one specific commodity and incremental costs in each age group. **(b)** Distribution of parameter estimates of total healthcare cost for geriatric IS patients with various combined diseases at different quantiles based on the quantile regression analysis. Quantile regression analysis revealed the influence of each combined disease on healthcare costs at different quantiles of healthcare costs. The middle line represents the average value and the semi-transparent area represents the 95% confidence interval. Three colors represent three age groups.

Spearman correlation analysis of the top 20 combined diseases and healthcare costs showed that the financial burden of combined diseases intensified with aging. In patients aged 60–69 years, incremental costs were weakly correlated with combined diseases such as ischemic heart disease, constipation, and renal failure. However, strong correlations were observed in patients aged ≥80 years, particularly pulmonary infection, hypoproteinemia, and anemia, which were most strongly associated with higher healthcare costs (Figure 5a). Quantile regression analysis further revealed how combined diseases influenced healthcare costs at various cost quantiles (Table 3). For anemia, arrhythmia, pulmonary infection, and hypoproteinemia, the cost burden increased at higher cost quantiles. In both 60–69 years and ≥80 years groups, pulmonary infection was significantly associated with incremental healthcare costs at the 10th, 25th, and 50th percentiles (60–69 years group: *τ* = 0.10, *β* = ¥4,424, adj. *p* < 0.01; *τ* = 0.25, *β* = ¥5,422, adj. *p* < 0.01; *τ* = 0.50, *β* = ¥8,627, adj. *p* < 0.01; *τ* = 0.90, *β* = ¥28,318, adj. *p* = 0.03; ≥80 years group: *τ* = 0.10, *β* = ¥5,953, adj. *p* < 0.01; *τ* = 0.25, *β* = ¥8,538, adj. *p* < 0.01; *τ* = 0.50, *β* = ¥13,810, adj. *p* < 0.01; *τ* = 0.75, *β* = ¥18,945, adj. *p* = 0.02; *τ* = 0.90, *β* = ¥41,369, adj. *p* = 0.37). Hypoproteinemia showed particularly high incremental costs at the 50th percentile in patients aged 60–69 years (*β* = ¥20,957, adj. *p* < 0.01), and 25th and 50th percentile in patients aged ≥80 years (*τ* = 0.25, *β* = ¥7,506, adj. *p* = 0.046; *τ* = 0.50, *β* = ¥12,962, adj. *p* < 0.01). In contrast, hyperlipidemia, hypertension, and hyperuricemia were associated with lower healthcare costs. The coefficient trajectory plot (Figure 5b) revealed that the patients aged ≥80 years experienced the largest shift from the 10th to the 90th percentile, with confidence intervals widening at higher percentiles. Sensitive analysis excluding the pandemic time from 2019 to 2020 further verified these results. The interpretation of the significant influence of cost from pulmonary infection and hypoproteinemia should note the variability from wide confidence intervals at high percentiles. The patterns of coefficient trajectory exhibited significant age-specific differences in the economic burden of ischemic stroke patients with combined diseases.

**Table 3:** Quantile regression analysis of combined diseases and healthcare costs at low, middle, and high-cost levels.

Combined diseases	60–69 years (*n* = 936) *β* (*τ*) (¥)					70–79 years (*n* = 934) *β* (*τ*) (¥)					≥80 years (*n* = 1,135) *β* (*τ*) (¥)				
	*τ* = 0.1	*τ* = 0.25	*τ* = 0.5	*τ* = 0.75	*τ* = 0.9	*τ* = 0.1	*τ* = 0.25	*τ* = 0.5	*τ* = 0.75	*τ* = 0.9	*τ* = 0.1	*τ* = 0.25	*τ* = 0.5	*τ* = 0.75	*τ* = 0.9
Atherosclerosis	−1,146	−1,540	−4,670	−8,079	−23,508	−258	−1,847	−3,717	−3,008	3,942	236	−483	−5,558	−15,280	−9,533
Hypertension	339	332	367	−59	−9,452	−1,060	−2,407	−1,232	−7,059	−12,165	−536	−1,047	−1,922	−4,901	−6,718
Hyperlipemia	−1,268	−1,330	−1,549	−4,463	−14,789	195	−957	−1,952	−3,539	−6,359	−935	−1,580	−1,852	−4,484	−24,377
Ischemic heart disease	−183	−197	−63	1,674	4,450	1,596	1,301	2,424	2,771	7,270	400	750	1,384	312	−5,002
Diabetes	1,129	1,772	2,278	796	4,185	687	757	900	−673	−6,977	1,222	1,839	1,417	2,457	8,980
Arrhythmia	−522	−17	−1,039	−5,132	−10,765	1,213	2,983	2,800	−1,054	−2,905	−100	585	2,201	4,096	4,250
Pulmonary infection	4,424^**^	5,422^**^	8,627^**^	17,972	28,318^*^	2,395	3,795	10,153^**^	16,237	18,068	5,953^**^	8,538^**^	13,810^**^	18,945^*^	41,369
Hypokalemia	−257	−505	−871	−2,308	−8,460	1,460	128	−1,218	−3,322	−7,546	2,244	820	−2,164	−7,646	−20,501
Reflux esophagitis	−301	53	−1,364	−6,360	−15,118	−1,559	−510	694	−652	−3,273	3,133	1,775	−1,454	−8,109	−13,704
Cysteinemia	−85	−822	−249	−3,434	−9,444	−1,409	−207	−371	−2,994	−7,520	−829	−1,193	−1,751	4,214	4,709
Prostatic hyperplasia	725	−312	−1,838	−3,396	−5,056	−365	−970	−505	−2,205	4,882	−1,658	−920	689	2,485	−19,812
Fatty liver	−1,738	−1,071	−2,720	−4,002	−5,791	723	−423	581	−2,429	5,593	−719	−2,233	−2,818	−8,196	−1,366
Lung mass	−781	−1,410	−3,900^*^	−5,622	−19,081^*^	761	−289	−1,139	−1,474	−3,404	−1,581	−3,032	−1,090	−260	−4,718
Renal failure	1,042	384	718	−1,276	966	3,036	3,404	4,795	5,229	8,221	1,113	1,955	740	−6,860	−17,363
Hypoproteinemia	2,067	4,573	20,957^*^	29,249	69,730	2,286	8,153	8,785	50,271	90,633	1,304	7,506^*^	12,962^**^	15,560	14,163
Anemia	918	382	5,870	11,827	7,771	334	265	−1,115	−4,765	368	463	1,706	3,728	9,597	22,084
Mental disorders	−1,263	−1,802	−1,782	−1,634	−7,822	−1,310	−2,832	−4,270	−5,935	−2,587	2,286	4,846	−1,962	481	−1,123
Peripheral neuropathy	−914	−1,633	−229	144	−9,256	487	891	1,536	1,909	5,771	−1,405	−1,705	−216	3,103	−4,175
Hyperuricemia	−1,102	−1,589	−2,675	−4,287	−5,995	−648	−1,866	−2,124	−6,630	−9,905	−2,741	−3,375	−5,156	−4,266	2,586
Constipation	−1,206	−1,127	−1,529	−2,357	−10,830	−1,180	−853	−3,753	−8,130	−14,865	−2,110	−470	−5,029	−2,236	−8,786

*β* (*τ*) denotes the quantile-regression coefficient at the *τ*-th quantile. ^*^adj. *p* < 0.05 and ^**^adj. *p* < 0.01.

## Discussion

The multicenter cross-sectional study of 3,005 geriatric ischemic stroke (IS) patients revealed significant epidemiological fluctuations during COVID-19 pandemic, with hospitalizations sharply declining in 2020 followed by a marked surge in 2021. This pattern likely reflects pandemic-related disruptions to chronic disease management (hypertension, dyslipidemia, atherosclerosis), delaying essential care (21). Concurrently, the decreasing proportion of patients aged ≥80 years during this period might indicate elevated out-of-hospital mortality among this vulnerable subgroup due to COVID-19 complications (21, 22).

Our large-sample analysis identified potential age-stratified patterns in stroke etiology, profile of combined diseases, and economic burden. The observed shift from large-artery atherosclerosis (LAA) predominance in patients aged 60–69 years toward cardioembolism (CE) in advanced age aligns with aging-related cardiac pathophysiology—particularly atrial fibrillation and structural heart disease. These findings provoked the need for age-tailored secondary prevention, including prioritizing anticoagulation in patients aged ≥80 years over antiplatelet therapy (23, 24). Moreover, the potential trend of longer hospital stays and higher in-hospital mortality underscore the vulnerability of patients aged ≥80 years, necessitating geriatric-focused stroke units.

In the analysis of comorbidities, we have identified several common comorbidities in the study population, whereas the composition of combined diseases varied in different age groups. The incidence of hyperlipidemia and diabetes was higher in patients aged 60–69 years, while in advanced ≥80 years group, there was a higher incidence of ischemic heart disease, arrhythmia, and pulmonary infection. The shift is clinically significant, as arrhythmia (predominantly atrial fibrillation) and post-stroke pneumonia (PSP) are major drivers of higher mortality and poor outcomes in IS patients (25–28). These findings underscore the need for age-tailored management, emphasizing proactive strategies like arrhythmia screening and pneumonia prevention protocols in the vulnerable advanced-old population. In addition, further analysis revealed clinically significant comorbidity clusters with critical implications for integrated care of geriatric IS patients. In addition, further analysis revealed clinically significant comorbidity clusters with critical implications for integrated care of geriatric IS patients. The cardio-metabolic cluster—dominated by hypertension, hyperlipidemia, and atherosclerosis—serves as the foundational driver of cerebrovascular and coronary pathology (29, 30). Hypertension induces endothelial dysfunction and arterial stiffness, leading to the acceleration of vascular aging and atherosclerosis (31). As is well known, lipid disorder promotes plaque formation, and there is a large amount of type 2 diabetes patients combined with ischemic heart disease (32, 33). This triad creates a self-perpetuating cycle of metabolic dysregulation and hemodynamic compromise, exacerbated by age-related chronic inflammation, oxidative stress, and impaired metabolic homeostasis (34–36). The high prevalence of cardio-metabolic cluster in geriatric IS patients underscores the need for integrated management strategies targeting shared risk factors (e.g., reasonable and individualized blood pressure control, statin therapy, rigorous blood glucose control) to mitigate multi-organ vascular complications. Future studies should explore whether these combined diseases synergistically worsen stroke outcomes or reflect broader senescence-related metabolic dysfunction.

Diabetes functioned as a pivotal mediator of multi-organ injury, bridging renal failure, peripheral neuropathy, and hypoproteinemia through microvascular and macrovascular pathways in the older population (37–39). Persistent hyperglycemia induces endothelial dysfunction and oxidative stress, accelerating renal microangiopathy and subsequent renal dysfunction (40). The proteinuria induced by diabetic nephropathy is directly correlated to the generation of hypoproteinemia (41). In the older population, age-related decline in organ reserve capacity particularly amplifies their vulnerability to multi-system dysfunction. Concurrently, metabolic disorders promote peripheral nerve demyelination and axonal degeneration, explaining the high prevalence of peripheral neuropathy (42). These findings indicate that geriatric IS patients require standardized monitoring for urinary albumin-to-creatinine ratio (UACR) and nerve conduction function, and it is of great significance to emphasize nutritional interventions and glycemic control in older population. Furthermore, we identified a malnutrition-infection-anemia triad with particularly severe manifestations in advanced age. Hypoproteinemia, reflecting chronic inflammation and malnutrition, compromises humoral immunity through impaired acute-phase protein synthesis, elevating pneumonia risk especially in dysphagic patients. Age-related sarcopenia depletes protein reserves while diminished respiratory reserve increases infection susceptibility. Concurrently, infection-induced cytokine storms (IL-6, hepcidin upregulation) disrupt erythropoiesis and iron metabolism, establishing anemia of chronic disease. This self-perpetuating triad significantly prolongs hospitalization and increases mortality risk.

Apriori algorithm was applied in the analysis of comorbidity clusters in the study. Apriori algorithm systematically generates strong, directional association rules through an iterative process. This method is innovative and intuitive, providing an effective tool for understanding complex disease co-occurrence patterns (43, 44). Compared to clustering analysis, aiming to grouping the population with similar comorbidity patterns into distinct categories, Apriori algorithm provides an effective tool for better understanding complex disease co-occurrence patterns (45). For instance, patients might be classified into cardiovascular-metabolic clusters or neuropsychiatric-degenerative clusters (46, 47). This method seeks to achieve a global classification, assigning each patient to a single category. The core distinction and advantage of the Apriori algorithm compared to clustering analysis lie in its departure from such global, exclusive partitioning (48). In clinical practice, a single condition such as obesity may be a key component of several different comorbidity patterns, a nuance that traditional clustering methods struggle to capture. By generating independent association rules, Apriori naturally allows a disease to be part of multiple comorbidity combinations, which more closely reflects clinical reality. Furthermore, most clustering methods require pre-specifying the number of clusters, whereas Apriori requires no such *a priori* assumption, automatically discovering all patterns that meet the criteria based on the data’s intrinsic associations (49, 50). Therefore, when the research goal is a clear, non-overlapping classification of patients or diseases, clustering analysis is suitable. However, when the need is to identify specific, overlapping comorbidity combinations that transcend categorical boundaries, the Apriori method is more insightful. Comorbidity network analysis treats diseases as nodes and their co-occurrence as edges to construct a macroscopic map of disease relationships (51). However, it is better suited for visualizing global, dyadic relationships and has limitations in identifying higher-order comorbidity patterns involving three or more diseases (52). The Apriori algorithm precisely addresses this gap by systematically mining these multivariate combinations and presenting them as precise, easily interpretable rules, which offers more direct guidance for clinical risk prediction and intervention strategies (53). In short, if the research objective is to explore the structural features and key nodes of the overall disease ecosystem, network analysis is more appropriate. Conversely, if the goal is to identify specific, high-confidence predictive signals for disease clusters, the Apriori algorithm holds a greater advantage. In summary, the Apriori algorithm, clustering analysis, and other comorbidity network analysis methods offer multiple complementary perspectives for multimorbidity research. Apriori focuses on mining specific and interpretable co-occurrence rules, clustering analysis is dedicated to the global classification of patients or diseases, and network analysis excels at depicting the macroscopic structure of disease relationships.

When it comes to healthcare costs, patients aged ≥80 years may have the highest costs compared to those in other groups, reflecting a convergence of biological vulnerability and resource-intensive care demands. It was considered to be correlated with poorer organ function, more types of combined diseases and more serious diseases, leading to longer hospital stay and more demand for all type of treatment (54). Interestingly, the overall costs among geriatric IS patients declined from 2018 to 2023, primarily driven by reduced drug expenditures, which contrasting with cost escalations in high-income countries. Hospitalization costs of ischemic stroke in our study were much lower than those reported in the US, where increased treatment costs for ischemic stroke have been driven by the popularization of advanced interventions like endovascular thrombectomy (55, 56). Even though, the economic burden of stroke in the US is supposed to rise significantly from 2020 to 2050, largely due to an aging population and the prevalence of cardiovascular risk factors (57). The discrepancy in cost trend may be due to recent healthcare policies in China, such as National Volume-Based Procurement (VBP) and the payment policy based on Diagnosis Related Groups (DRG) and, leading to the reduction in the cost of medical drugs and materials and adopting cost-saving strategies in ischemic stroke healthcare (58, 59). However, this assumption would require a formal interrupted time-series analysis or a comparison with control hospitals, which falls outside the primary scope of the present research. Future studies utilizing such methods will be planned to more comprehensively assess the changes in healthcare costs before and after the implementation of these policies. Additionally, the patients who were aged ≥80 years incurred higher costs compared to younger patients, which were not in line with findings in Australia, where ischemic stroke patients aged 70–79 had the highest treatment costs, possibly due to the variabilities of demographic distribution, lengths of stay and distribution of combined diseases (54). Although the length of stay (LOS) was adjusted for in our regression model, it remains a critical factor influencing healthcare costs. Longer hospital stays are usually associated with higher medical resource utilization, including increased consumption of nursing care, medications, diagnostic tests, and other hospital services, all of which may contribute to higher overall costs. It is important to acknowledge that LOS independently drives healthcare costs, particularly in patients with more severe conditions and complex treatment needs. While we adjusted for LOS in our analysis, its independent association with increased expenditures warrants further consideration.

After the in-depth analysis of comorbidity correlation network, it is indicated that pulmonary infection, hypoproteinemia, and anemia might be the key combined diseases associated with high healthcare costs, particularly in patients aged ≥80 years (54, 60). Notably, pulmonary infection and hypoproteinemia demonstrated a strong positive association with significantly higher medical expenditures. Factors contributing to the high costs in patients with these conditions could include severer underlying diseases, complex comorbidities, prolonged length of hospitalization, utilization of high-grade antibiotics and albumin, as well as expensive supplements for nutritional deficiencies (61–63). A systematic review revealed that length of stay, as the primary driver of costs, is often extended by complications like infection, malnutrition, or hypoalbuminemia, thereby significantly increasing overall medical expenses (64). Consequently, these findings indicated that the integrated management of pulmonary infection-hypoproteinemia-anemia cluster might be a critical strategy for reducing healthcare costs among geriatric IS patients in China.

The study is a large, multicenter cross-sectional study, aiming to reveal real-world profile of combined diseases and cost data of geriatric IS patients at different age stratification. Despite overall cost reductions from policy reforms, aging demographics threaten future economic sustainability. Our findings collectively advocate for a three-tiered approach to optimize IS treatment: (1) Primary prevention through intensive management of modifiable vascular risk factors, notably hypertension and dyslipidemia, to reduce incident stroke and downstream complications; (2) Secondary intervention via protocolized bundle management of the high-impact malnutrition-infection-anemia triad (serum albumin monitoring, early dysphagia screening, and immunonutrition) to mitigate cost-driving complications; and (3) Policy enhancement requiring VBP policies and DRG system refinements that explicitly account for geriatric complexity through combined diseases-adjusted reimbursement coefficients, ensuring resource allocation aligns with the multidimensional needs of aging stroke populations.

Several limitations merit acknowledgment while charting future research priorities. First of all, the cross-sectional design inherently precludes definitive causal inference between combined diseases and healthcare expenditures. Secondly, focusing on middle-high-income tertiary hospitals from a single region introduces potential sampling bias, which may restrict the generalizability of the results to other regions of China, particularly rural or under-resourced areas. The differences in healthcare delivery across hospital tiers and socioeconomic may influence the comorbidity profiles and cost structures observed in this study, which require further exploration. Thirdly, the study focused on economic outcomes, without a comprehensive assessment of clinical and functional metrics, limits the full interpretation of comorbidities “real-world” burden. Our retrospective data, drawn from inpatient records, lacked crucial parameters such as stroke severity scores (e.g., NIHSS), detailed acute management data, and, critically, post-stroke functional status (e.g., 90-day mRS), long-term disability levels, and post-discharge mortality. While we did assess in-hospital mortality, the absence of this longitudinal data makes it challenging to fully correlate the identified comorbidity clusters with long-term recovery and survival. Therefore, while our study successfully highlights comorbidities associated with high inpatient costs, a prospective cohort study, which we plan to conduct, is necessary to determine how these clusters influence long-term functional independence and patient-centered outcomes. We focused exclusively on the top 20 combined diseases risks overlooking rare yet clinically significant condition. Based on the limitations, we are planning to collect data from various regions to ensure that the results are scalable. Critically, we are going to carry out prospective cohort study to further confirm the causal relationship and assess how these strategies impact the long-term sustainability of stroke care, particularly for the older adults to forecast healthcare system sustainability amid rapid population aging. Additionally, the possible influence of volume-based procurement and DRG policies were not formally verified by a formal interrupted time-series analysis due to limitations in the available data. Further studies were needed to examine the changes in healthcare costs before and after the implementation of the VBP and DRG policies to provide a more robust evaluation of the effect.

## Conclusion

In this study, we have explored how aging reshapes ischemic stroke pathophysiology, comorbidity networks, and economic burdens. Key findings reveal: (1) a transition from large-artery atherosclerosis to cardioembolic dominance beyond age 80; (2) three high-impact comorbidity clusters, cardio-metabolic, diabetes-mediated multi-organ injury, and malnutrition-infection-anemia, with distinct age-specific prevalence; (3) pulmonary infection, hypoproteinemia, and anemia as potential cost drivers, particularly in patients ≥80 years. The observed significant cost reduction from 2018 to 2023 was assumed to be attributed to the national healthcare reforms, yet this speculation requires further verification by formal interrupted time-series analysis. Implementation of precision geriatric protocols, including integrating comorbidity network screening, early diabetes complication mitigation, and targeted triad management, promises to optimize both clinical outcomes and healthcare economics for aging stroke populations.

## Data Availability

The raw data supporting the conclusions of this article will be made available by the authors, without undue reservation.
